# Immune Dysregulation in HIV and COVID‐19 Co‐infection: Therapeutic Implications

**DOI:** 10.1002/iid3.70164

**Published:** 2025-03-26

**Authors:** Maryam Nejabat, Mohammad Motamedifar, Saeid Amirizadeh Fard, Mohammadreza Heydari, Soudabeh Bemani

**Affiliations:** ^1^ HIV/AIDS Research Center, Institute of Health Shiraz University of Medical Sciences Shiraz Iran; ^2^ Department of Bacteriology and Virology, Shiraz Medical School Shiraz University Medical Science Shiraz Iran; ^3^ Gastroenterohepathology Research Center Shiraz University of Medical Sciences Shiraz Iran; ^4^ Shiraz HIV/AIDS Research Center (SHARC) Shiraz University of Medical Sciences Shiraz Iran

**Keywords:** COVID‐19, cytokine, HIV infections, interleukin

## Abstract

**Background:**

Co‐infection with HIV and SARS‐CoV‐2 presents a complex clinical picture. Deciphering the immune response in this population, particularly the role of cytokines underlying immunopathogenesis could elucidates the development of targeted therapeutic interventions.

**Methods:**

This prospective, two‐stage study enrolled 75 individuals with HIV diagnosed with COVID‐19 (case group) and 25 individuals from the general population infected with SARS‐CoV‐2 only (control group). COVID‐19 diagnosis followed World Health Organization guidelines. Plasma cytokine levels were measured using a cytokine bead array.

**Results:**

The case group skewed slightly females (61.2% vs. 42.9% female in the control group) an average age of 3 years older (44.13 years vs. 40.86 years). Importantly, all the case group participants had mild complications, while a significant majority (88.1%) in the control group experienced severe complications. The control group displayed a substantially higher IgM titer 963 IU/mL compared to only 39.3 IU/mL in the case group. The control group had significantly higher levels of IL‐6, IL‐10, IFN‐γ, TNF‐α compared to the case group.

**Conclusion:**

This study suggests a potentially distinct immune response in HIV‐positive patients when infected with SARS‐CoV‐2. Elucidating these differences could lead to the development of more effective treatment strategies for this vulnerable population.

## Summary

- This study investigated the correlation between plasma levels of interleukins in PLWH with COVID‐19 compared to the general population.
- There were significantly lower levels of IL‐6, IL‐10, IL‐17A, INFγ, and TNF‐α in PLWH compared to the control group in both acute and recovery phases of COVID‐19.
- These findings suggest a potential link between HIV infection and altered immune response to SARS‐CoV‐2.

## 1 Introduction

The COVID‐19 pandemic has posed unprecedented challenges to global healthcare systems, particularly for individuals with pre‐existing immunocompromised conditions, such as those infected with Human Immunodeficiency Virus (HIV) [1]. The interplay between HIV and SARS‐CoV‐2 presents a multifaceted clinical dilemma, underscoring the necessity for a deeper understanding of the immunological dynamics and molecular mechanisms underpinning this dual affliction [2]. As the pandemic continues to evolve, the complexity of managing co‐infected patients has become increasingly apparent, highlighting a critical gap in our understanding of how these two viral infections interact and impact the immune system.

HIV, a retrovirus that specifically targets and depletes CD4^+^ T cells, significantly impairs the immune response of the body. This immunosuppression leaves individuals highly susceptible to a range of opportunistic infections and malignancies [3]. The advent of SARS‐CoV‐2, the virus responsible for COVID‐19, has exacerbated the health challenges faced by those living with HIV. The co‐infection of HIV and SARS‐CoV‐2 has been associated with more severe clinical outcomes, including increased mortality rates and a higher risk of developing critical illness compared to the general population [4]. These observations underscore the urgency of investigating the immunological interplay between these two pathogens to inform better management strategies and therapeutic interventions.

This study addresses a critical gap in understanding the immunological interplay between HIV and SARS‐CoV‐2 by exploring plasma cytokine profiles in co‐infected individuals. Prior research has largely focused on single infections, leaving the immune dynamics of dual infections underexplored. By comparing cytokine levels such as IL‐6, IL‐10, TNF‐α, and IFN‐γ between HIV‐positive patients with COVID‐19 and the general population with COVID‐19, the study provides insights into the altered immune responses in co‐infected individuals. This contributes to a nuanced understanding of how immunosuppression due to HIV impacts the progression and recovery from SARS‐CoV‐2 infection, helping to inform tailored therapeutic strategies.

Central to understanding the immune response in co‐infected individuals is the role of cytokines, particularly interleukins, which are critical mediators of the immune system function. Interleukins are involved in various aspects of immune regulation, including inflammation, cell proliferation, and differentiation. This study focuses on several interleukins, including interleukin 6 (IL‐6), IL‐17A, IL‐17F, IL‐10, and IL‐22, and their interplay with interferon gamma (IFN‐γ) and tumor necrosis factor (TNF). IL‐6 has gained prominence for its involvement in the cytokine storm observed in severe COVID‐19 cases, a phenomenon that contributes to the development of acute respiratory distress syndrome (ARDS) and subsequent organ damage [5]. The overproduction of IL‐6 is thought to drive systemic inflammation and exacerbate disease severity, making it a crucial target for therapeutic intervention.

IL‐17A and IL‐17F, members of the IL‐17 family, are known for their role in promoting pro‐inflammatory responses [6]. These interleukins have been implicated in the pathogenesis of various autoimmune and inflammatory disorders. In the context of HIV and SARS‐CoV‐2 co‐infection, their levels could reflect the extent of immune activation and inflammation. IL‐10, on the other hand, is renowned for its anti‐inflammatory properties and its role in maintaining immune homeostasis [7]. It acts as a counterbalance to pro‐inflammatory cytokines, preventing excessive tissue damage and modulating the overall immune response. IL‐22 is another key interleukin with a protective role in mucosal immunity, contributing to tissue repair and regeneration during infections [8].

The study also aimed to explore the role of IFN‐γ and TNF in the context of co‐infection. IFN‐γ is a pivotal cytokine in antiviral defense, orchestrating the immune response against viral pathogens such as SARS‐CoV‐2. It enhances the ability of immune cells to combat infections and modulates the activity of other cytokines [9]. TNF, a pleiotropic cytokine, is central to inflammation and immune regulation, and its dysregulation is associated with a range of inflammatory diseases [10]. Understanding the levels and interactions of IFN‐γ and TNF in the setting of HIV and SARS‐CoV‐2 co‐infection could provide valuable insights into the mechanisms driving disease progression and immune dysfunction.

Given the complex nature of immune dysregulation observed in people living with HIV (PLWH) who are also infected with SARS‐CoV‐2, it is crucial to elucidate the specific relationships and potential interactions between these cytokines. Such an understanding could inform the development of targeted therapeutic strategies and enhance clinical outcomes. By shedding light on the immunological interplay between these factors, the research seeks to contribute to the growing body of knowledge and support the design of more effective treatment protocols.

Through this exploration, we aimed to advance our understanding of the immunological landscape in co‐infected patients and offer insights that could lead to improved management and therapeutic approaches for this challenging patient population.

## 2 Materials and Methods

### 2.1 Study Design and Samples

We conducted a prospective two‐stage study on 75 PLHIV treated with retroviral drugs who were infected with SARS‐CoV‐2, as the case group, and 25 individuals from the general population infected with SARS‐CoV‐2, as the control group. The study participants were selected from the Referral Behavioral Counseling and Modification Center in Shiraz, Fars, Iran, from October 2021 to March 2022. Diagnosis of COVID‐19 infections was established following the criteria stipulated by the World Health Organization guidelines, comprised of serological and molecular findings. Exclusion criteria for the study encompassed participants with discrepancies or incomplete documentation of drug side effects in their medical records. Vaccinated patients were excluded from the study to prevent bias. Pregnant women were also not included. All participants signed informed consent forms before their involvement in the study, which received approval from the Ethics Committee of Shiraz University of Medical Sciences with the code of IR.SUMS.REC 1400.842, Shiraz, Iran.

Based on the NIH guideline participants were divided into two groups, the mild complication group and the severe complication group. The severe group included those who had respiratory rate > 30 breaths per minute, SpO₂ < 94% on room air at sea level, a ratio of arterial partial pressure of oxygen to fraction of inspired oxygen (PaO₂/FiO₂) of < 300 mmHg that severe patients were treated with Remdesivir [11]. Those patients who had any of common signs and symptoms (e.g., fever, cough, sore throat, malaise, headache, muscle pain, nausea, vomiting, diarrhea, loss of taste, and smell) without shortness of breath, dyspnea, or abnormal chest imaging were considered as the mild complication group.

We considered the acute phase of the disease when patients visited the hospital due to clinical symptoms, antibody and PCR tests were positive. Also, 4 months after the acute phase, when the immune response from a COVID‐19 infection usually tamps, is considered the recovery phase [12].

Nasal swab samples were collected in viral transport media (VTM) and stored at 4°C for up to 24 h before being frozen at −80°C for further analysis.

Blood samples were collected from a peripheral vein using vacutainers containing EDTA. A volume of 5 mL of blood was drawn from each patient in a fasting state immediately before transfusion. The collected samples were centrifuged at 3000 rpm for 10 min, and the plasma was separated and stored at −80°C without the addition of any preservatives until analysis.

Plasma cytokine levels (IL‐6, IL‐10, IL‐17A, IL‐17F, IL‐22, tumor necrosis factor alpha [TNF‐α], and interferon gamma) were evaluated in both acute and recovery phases.

### 2.2 SARS‐CoV‐2 IgM and IgG Antibody Detection

The IgM and IgG antibodies against SARS‐CoV‐2 in plasma specimens were detected using IgG and IgM kits (Ideal Tashkhis Co., Tehran, Iran), following the manufacturer's guidelines. The recombinant antigens include the nucleoprotein and spike protein of SARS‐CoV‐2. For both IgG and IgM kits, a 0.9 cut‐off index was applied, where results ≥ 0.9 were considered reactive (positive), and results < 0.9 were categorized as nonreactive (negative).

### 2.3 RNA Extraction

To extract the viral RNA from nasopharyngeal samples, we utilized TRIzol reagent (Life Technologies, CA, USA) based on the manufacturer's protocol. Subsequently, one microgram of total RNA was utilized for cDNA synthesis, employing random hexamer and oligo (dT) primers, through RevertAid Reverse Transcriptase (Fermantas, Lithuania), following the manufacturer's instructions, in a total reaction mix volume of 20 µL.

### 2.4 PCR Assay

SARS‐CoV‐2 PCR assay was performed using a Rotor‐Gene Q (Qiagen) platform containing 2 μg template cDNA, Ampliqon real Q Plus 2× Master Mix for probe without Rox (Ampliqon, Herlev, Denmark), 0.4 μM of each primer and 0.25 μM of probe in 25 µL final volume. The PCR employed the following thermal settings: 15 min at 95°C, followed by 45 cycles of 30 s at 95°C, 45 s at 59°C, and 30 s at 72°C. Negative controls and standards were included in every assay run. The number of SARS‐CoV‐2 RNA transcripts (copies/mL) of clinical samples was determined based on the standard curve. Potential interference between primers/probes for the target (SARS‐CoV‐2) was assessed by running serial dilutions of two clinical samples with confirmed high viral loads of SARS‐CoV‐2.

### 2.5 Cytokine Assay

The concentration of plasma cytokines (IL‐6, IL‐10, IL‐17A, IL‐17F, IL‐22, tumor necrosis factor alpha [TNF‐α], interferon gamma was quantified LEGENDplex MU Th17 Panel (7‐plex) w/FP V03 (BioLEGEND, UK), according to the manufacturer's instructions.

### 2.6 Statistical Analysis

IBM SPSS (version 26.0) was used for data analysis. The normality of data distribution was assessed by descriptive statistics (skewness, Kurtosis, and standard deviation [SD]). Parametric, non‐parametric, and qualitative data are expressed as mean ± SD, mean (95% confidence interval), or frequency (percentages), respectively. Between‐group differences were determined using the independent sample *t*‐test for parametric variables, Mann–Whitney *U* or Kruskal–Wallis tests for non‐parametric parameters, and Chi‐square test for categorical variables. A two‐sided *p*‐value < 0.05 was considered significant. We performed adjustments by ANCOVA both in the acute and recovery phases for sex and age.

## 3 Results

### 3.1 Characteristics of the Study Participants

The case group consisted of 55 (61.2%) women and 35 (38.8%) men. For the control group, there were 18 (42.9%) females and 24 (57.1%) males.

The mean age for the case group was 44.13 (26–69 years), and for the control group was 40.86 (22–62 years) (Table 1).

**Table 1:** The participants' demographic features.

	Gender (mean)	Age, Mean (Range)	Complication, Severe Mild	Death	SARS COV‐2 IgM	SARS COV‐2 IgG
Case	Male (38.8%) Female (61.2%)	44.13 (26–69)	0.0%, 100%	No	39.3 ± 52.6	6.9 ± 4.1
Control	Male (57.1%) Female (42.9%)	40.86 (22–62)	88.09%, 11.91%	No	963 ± 313.4	18.9 ± 12.2

### 3.2 Plasma Cytokine Profiles

To estimate the role of cytokines in individuals PLWH and infected with SARS‐CoV‐2, we measured their plasma levels by cytokine bead array.

As for the complications of the disease in the case group, all of them suffered mild complications.

Also, in the control group, 88.09% of them had severe and 11.91% had mild complications. There was no expiration in all groups.

Based on the ELISA test, it was found that SARS‐CoV‐2 IgM titer in the control group was higher than in the case group (963 ± 313.4 and 39.3 ± 52.6, respectively) (Table 1).

**Figure 1:**
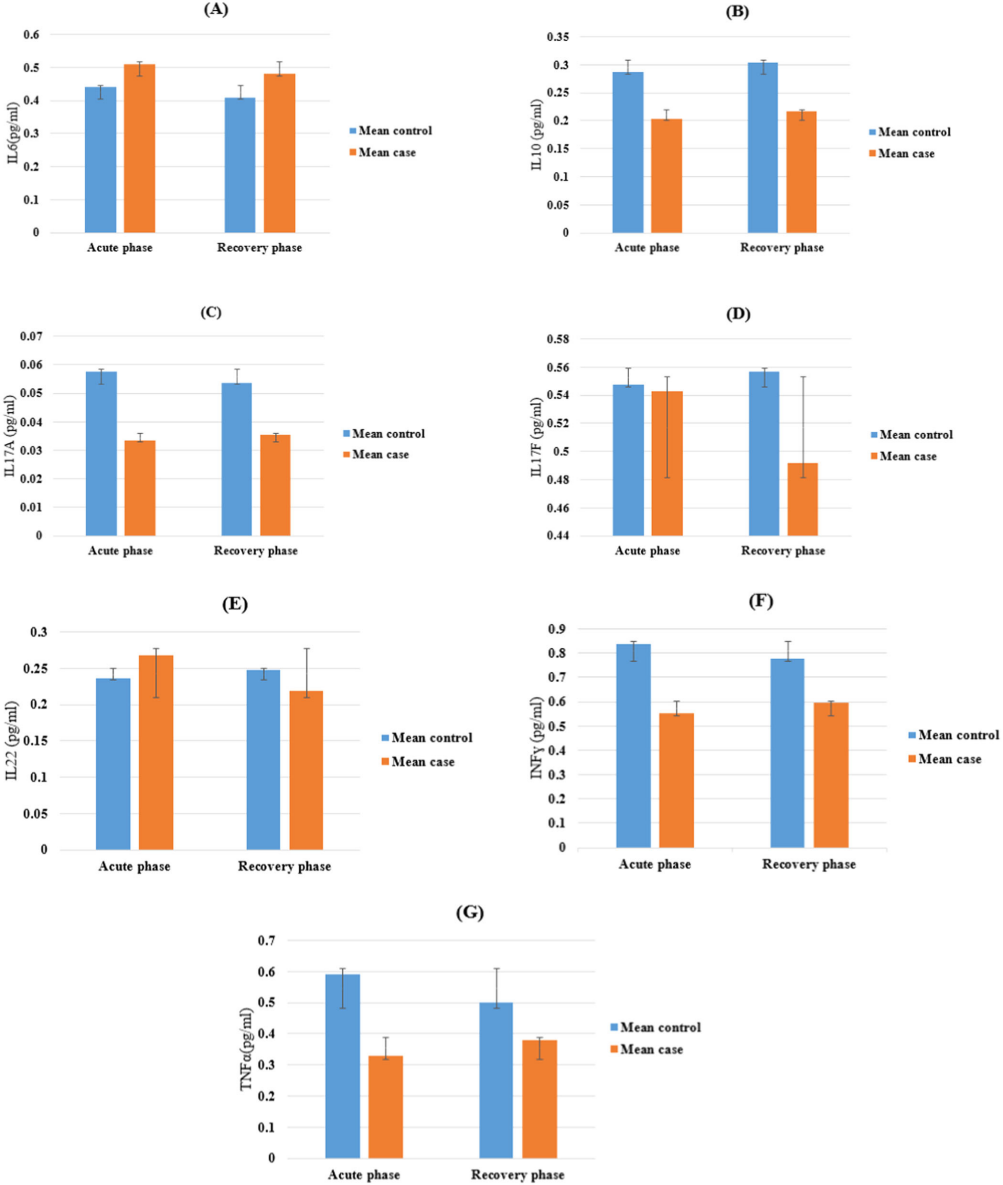
Plasma cytokine levels of patients at the acute and recovery phases of SARS‐CoV‐2 infection were measured by Flow cytometry bead array. (A) IL‐6 concentration, (B) IL‐10 concentration, (C) IL‐17A concentration, (D) IL‐17F concentration, (E) IL‐22 concentration, (F) INFγ concentration, (G) TNF‐α concentration. Abbreviations: IFN, interferon; IL, interleukin.

After statistical analysis, the levels of IL‐6, 17 A, 17 F, 10, 22, IFN‐γ, and TNF in the plasma of HIV‐positive patients' co‐infection with SARS‐CoV‐2 and the general population of COVID‐19 are shown in Figure 1 and Table 2.

**Table 2:** Cooperation of cytokines plasma concentration in the case and control groups in to time periods.

Variable	Case	Control	*p*‐value between groups	*p*‐value adjust
IL6				
Acute phase	0.44 ± 0.13	0.51 ± 0.26	0.11	
Recovery phase	0.41 ± 0.01	0.48 ± 0.27	< 0.001	< 0.001*
*p*‐value within group	0.098	0.197		
Diff_IL6	−0.024 (CI: −0.536/0.046)	0.058 (CI: −0.329/0.369)	0.083	
IL10				
Acute phase	0.203 ± 0.08	0.287 ± 0.09	< 0.001	
Recovery phase	0.21 ± 0.05	0.30 ± 0.15	< 0.001	0.008*
*p‐*value within group	0.127	0.484		
Diff_IL10	0.012 (CI: −0.003/0.029)	0.017 ± (CI: −0.031/0.065)	0.869	
IL17A				
Acute phase	0.033 ± 0.02	0.057 ± 0.02	< 0.001	
Recovery phase	0.035 ± 0.01	0.053 ± 0.02	< 0.001	< 0.001*
*p*‐value within group	0.570	0.528		
Diff_IL17A	0.001 ± (CI: −0.004/0.008)	−0.003 (CI: −0.015/0.008)	0.406	
IL17F				
Acute phase	0.542 ± 0.39	0.547 ± 0.17	0.918	
Recovery phase	0.492 ± 0.197	0.557 ± 0.189	0.074	0.152
*p*‐value within group	0.231	0.827		
Diff_IL17F	−0.050 ± (CI: −0.133/0.032)	0.009 ± (CI: −0.766/0.966)	0.380	
IL22				
Acute phase	0.267 ± 0.144	0.236 ± 0.113	0.220	
Recovery phase	0.218 ± 0.110	0.247 ± 0.105	0.169	0.048*
*p*‐value within group	< 0.001	0.636		
Diff_IL22	−0.048 (CI: −0.074/−0.021)	0.011 (CI: −0.035/0.057)	0.019	
INF				
Acute phase	0.551 ± 0.413	0.838 ± 0.345	< 0.001	
Recovery phase	0.594 ± 0.323	0.778 ± 0.472	0.026	0.293
*p*‐value within group	0.361	0.409		
Diff‐INF	0.043 (CI: −0.050/0.136)	−0.059 (CI: −0.202/0.084)	0.225	
TNF‐α				
Acute phase	0.329 ± 0.183	0.590 ± 0.360	< 0.001	
Recovery phase	0.378 ± 0.152	0.501 ± 0.307	0.018	0.913
*p*‐value within group	0.015	0.056		
Diff‐TNF‐α	0.049 (CI: 0.009/0.088)	−0.089 (CI: −0.180/0.002)	0.007	

In this study, the plasma level of IL6 and IL10 in both acute and recovery phases in the control group compared to the case group was significantly higher (*p* < 0.001), while the plasma levels of IL‐17F between the case and control group were not significantly different (*p* = 0.918, *p* = 0.074 for acute and recovery phase, respectively).

On the other hand, IL‐17A concentration was increased in the control group compared to the case group (*p* < 0.001).

There was no significant variation in the IL22 plasma level in both groups for both phases (*p* = 0.220, *p* = 0.169 for the acute and recovery phases, respectively). However, after doing adjustments (to age and sex), both in the acute and recovery phases, we observed a significant increase in the control group compared to the case group (*p* = 0.048).

As for INFγ and TNF‐α concentration, there was a significant elevation in both phases for the control group compared to the case group (*p* = 0.001 for the acute phase, *p* = 0.026 and *p* = 0.018 for INFγ and TNF‐α in the recovery phase, respectively).

## 4 Discussion

The novel coronavirus exhibits a high level of contagion, resulting in a substantial 2% mortality rate [13]. Individuals infected commonly manifest symptoms, including fever, fatigue, respiratory complications, and gastrointestinal issues. In severe instances, the virus may precipitate acute respiratory distress syndrome, septic shock, multiorgan failure, or fatality [14].

Nevertheless, owing to the swift propagation of SARS‐CoV‐2 and the absence of specific remedies, the knowledge deficit concerning its influence on the immune system remains a critical concern. Previous investigations highlight the pivotal role cytokine storms play in severe COVID‐19 cases [15, 16, 17].

HIV‐induced immunosuppression, particularly the depletion and dysfunction of CD4^+^ T helper cells, may attenuate the hyperinflammatory response associated with severe COVID‐19, potentially reducing cytokine storms and tissue damage. This paradoxical effect raises the possibility of leveraging targeted immunosuppressants, such as IL‐6 inhibitors, to manage severe COVID‐19 in non‐HIV‐infected individuals. Further research is needed to confirm this phenomenon in larger cohorts and to elucidate the mechanisms of interaction between HIV‐associated immunosuppression and SARS‐CoV‐2 infection. These findings could inspire novel therapeutic strategies for controlling hyperinflammatory conditions while preserving essential immune functions.

Comorbidities like diabetes and cardiovascular diseases are known to exacerbate COVID‐19 outcomes, as both conditions are associated with chronic inflammation and immune dysregulation. In the context of HIV and COVID‐19 co‐infection, these comorbidities could further amplify immune dysfunction, potentially worsening disease severity. Chronic conditions may also interact with the dysregulated cytokine response observed in this study, such as elevated IL‐6 and TNF‐α, creating a complex immunological environment. Future studies should stratify participants based on comorbidities to better understand their impact on disease progression and immune responses in co‐infected patients.

Regarding the sex distribution in our study, while COVID‐19 incidence was higher among females in the case group compared to the control group, the overall sex ratio remained balanced, with no statistically significant difference between the two groups. Furthermore, our findings did not reveal significant differences in underlying comorbidities (e.g., hypertension, diabetes) between the case and control groups in relation to COVID‐19 infection. This could be attributed to the potential impact of different combination drug regimens used by the participants.

Research by Wen et al. has asserted that in COVID‐19 patients, T‐cell and B‐cell interactions stimulate T cells to produce IL‐2, leading to escalated B‐cell proliferation. This may elucidate the connection between T and B activators expressing IL‐6, TNF‐α, and IL‐1β [18].

Based on evidence, Th2 cells secrete IL‐4, IL‐6, and IL‐10 and enhance humoral immune responses [18].

In our study, the plasma level of IL6 and IL10 in both acute and recovery phases in the control group compared to the case group was significantly higher. In the study with a similar target population [19], the levels of inflammatory cytokines between the one with HIV infection and the group with HIV/SARS‐CoV‐2 co‐infection were compared. They found that IL‐6 did not differ significantly. On the contrary, they saw a difference in terms of TNF‐α levels (higher in HIV/SARS‐CoV‐2 than in the HIV group). Of note, similar to this study, IL‐6 was lower in the HIV/SARS‐CoV‐2 ‐co‐infected individuals than in COVID‐19 patients.

In the study conducted by Merza et al. [20], the concentration of IL‐6 was significantly higher in moderate COVID‐19 and severe cases of COVID‐19 groups compared to the control and recovered groups, which confirmed our results.

More recent studies indicated that Th1 cells secreted IL‐2, IFN‐γ, and TNF‐α and participated in the cellular immune response [18].

We demonstrated that TNF‐α concentration had a significant reduction in both phases for the PLWH co‐infected COVID‐19 compared to the control group, which is similar to Vergori et al.'s findings [19]. The quality of T‐cell responses in PLWH is often inferior to that of healthy donors, with a notable reduction in polyfunctional T‐cell responses, including TNF‐α and IL‐17A production [21, 22].

While some PLWH can mount a robust immune response, the overall cytokine profile suggests a compromised ability to produce key inflammatory cytokines [23].

We found that INFγ concentration had a significant elevation in both phases for the control group compared to the case group although in the Merza's study, it was shown that there were no significant differences in various times [20]. Conversely, in Rodriguez et al.'s study, a decreasing trend was observed in IFN‐ɣ and IL‐6 from early admission to the hospital through recovery during the weeks of the study [24]. The alteration in the responses could be due to different target populations.

Current findings indicate a substantial difference in the mean concentration of plasma interleukin‐17A between the control and case groups, being consistently higher in the control group during both phases. The nonsignificant differences in IL‐22 and IL‐17F levels between the case and control groups raise interesting questions about their roles in co‐infection. IL‐22 is crucial for mucosal immunity and tissue repair, but its lack of significant variation might suggest that it is not a key player in the immune dysregulation associated with HIV and SARS‐CoV‐2. Similarly, IL‐17F, a pro‐inflammatory cytokine, showed no significant changes, indicating that other members of the IL‐17 family, like IL‐17A, may have a more prominent role. These findings suggest that the immune response to co‐infection is selective, with some cytokines being more actively involved than others. Further research should investigate why certain cytokines remain stable and whether they contribute to immune homeostasis in these patients. However, no significant difference was observed in interleukin‐17F concentration between plasma levels of the control and case groups. Furthermore, our study revealed a noteworthy decrease in interleukin‐22 concentration in the case group during both acute and recovery phases. After adjusting for age, gender, and acute phase of the disease during recovery, we noted a significant increase in plasma interleukin‐22 concentration in the control group compared to the case group.

The SARS‐CoV‐2 virus binds to alveolar epithelial cells, activating both innate and adaptive immune responses and triggering the release of numerous cytokines. In our study, we studied the cytokine response of patients during the acute and recovery stages of coronavirus disease [25].

The findings of this study underscore the importance of monitoring cytokine profiles, particularly IL‐6, IL‐10, TNF‐α, and IFN‐γ, in co‐infected patients. Clinical management could benefit from targeted therapies aimed at modulating cytokine levels, such as IL‐6 inhibitors, to mitigate systemic inflammation and reduce disease severity. Future research should focus on the long‐term immune responses in co‐infected individuals, particularly after recovery, to identify potential risks for chronic inflammation or immune exhaustion. Additionally, investigating the impact of comorbidities, vaccination status, and antiretroviral therapy regimens on cytokine responses could help develop more personalized treatment approaches.

Earlier studies suggest that SARS‐CoV‐2 infections significantly elevate cytokine levels, inducing a cytokine storm that disrupts immune function and vital organ processes [26]. Conversely, in individuals with HIV, findings indicate that those recovering from SARS‐CoV‐2 infection enhance their antiviral capabilities within a specific timeframe, resulting in a relatively active immune state [27].

Although there is a lack of research on a similar target population, PLWH, and most studies on the general population did not follow different groups, there was insufficient data to compare our results with others. The relatively small sample size could affect the statistical results. Also, the variation in cytokine levels between studies could be due to differences in sampling time, the specific stage of the coronavirus life cycle in the host, and differences in disease severity. Further studies are necessary to address these gaps in understanding. The study's exclusion of vaccinated participants limits the generalizability of its findings. Emphasizing the need for larger, multicenter studies that include diverse populations and vaccinated individuals to validate and expand upon these findings. Addressing these gaps could provide more comprehensive insights into the immunological landscape of HIV and SARS‐CoV‐2 co‐infection. However, limited research exists on cytokines in patients post‐recovery from SARS‐CoV‐2 infection, especially in the PLWH population. Further investigations are necessary to address these gaps in understanding.

## 5 Conclusion

This study reveals a potentially distinct immune response in HIV‐positive patients co‐infected with SARS‐CoV‐2, suggesting that their immune systems may function differently compared to those without HIV. These findings highlight the importance of further investigating unique immune mechanisms, including understanding the importance of cytokines and immune cell subsets and their changes during different stages of COVID‐19 in this population. By elucidating these differences, researchers can develop more targeted and effective treatment strategies tailored to the specific needs of PLWH. Such advances could significantly improve clinical outcomes and quality of life for this vulnerable group. Furthermore, this study opens new avenues for understanding immune responses in the context of co‐infections and contributes to broader insights into immunology and the management of infectious diseases.

## Author Contributions

**Maryam Nejabat:** conceptualization, project administration, resources, writing – original draft, data curation, writing – review and editing. **Mohammad Motamedifar:** resources, writing – review and editing. **Saeid Amirizadeh Fard:** methodology, resources, writing – original draft, writing – review and editing. **Mohammadreza Heydari:** funding acquisition, writing – review and editing. **Soudabeh Bemani:** writing – review and editing.

## Data Availability

The authors have nothing to report.
